# The PAPER heuristic: a structured cognitive aid to reduce intrinsic cognitive load in early clinical reasoning training

**DOI:** 10.1186/s12909-026-09639-0

**Published:** 2026-06-16

**Authors:** Marius Gebauer, Frank Herbstreit, Thorsten Brenner, Sven Benson, Cynthia Szalai

**Affiliations:** 1 Institute for General Medicine, Medical Faculty, University of Duisburg-Essen, Essen, Germany; 2 Department of Anaesthesiology and Intensive Care Medicine, University Hospital Essen, University Duisburg-Essen, Essen, Germany; 3 Institute for Medical Education, C-TNBS, University Duisburg-Essen Faculty of Medicine, Essen, Germany; 4 Faculty of Medicine, University Duisburg-Essen, Essen, Germany

**Keywords:** Clinical reasoning, Cognitive load, Medical education, Simulation-based learning, Undergraduate medical education, Cognitive aid, Non-technical skills, Delphi method, Emergency medicine education, Curriculum timing

## Abstract

**Background:**

Clinical reasoning is a key competency in undergraduate medical education but is often taught implicitly. Structured approaches such as acronyms may help support students in developing this skill, especially in complex or high-stakes situations. The acronym PAPER (Patient, details of Acute situation, relevant Past medical History, Estimate the situation, Recommendations) was developed to provide a simple and structured framework to support clinical reasoning.

**Methods:**

The acronym was developed through a three-round Delphi process with clinical experts in emergency medicine, anaesthesiology, and trauma surgery. It was piloted in a single-blind, simulation-based study with 290 medical students in either their fourth clinical semester or final year. Students were randomised to either an intervention group, which received a 20-minute introduction to clinical reasoning and the acronym before simulation training, or a control group, which received standard simulation alone. The outcomes included clinical performance, nontechnical skills, and cognitive load, which were assessed via blinded raters and validated self-report questionnaires.

**Results:**

No significant differences in clinical performance were found between the intervention and control groups. However, among fourth-semester students, the intervention group reported a significantly lower intrinsic cognitive load than did the control group. The final-year students generally reported better scores in teamwork and decision-making but reported lower germane cognitive loads than their junior peers did. The effect of the intervention appeared to be influenced by the students’ training stage.

**Conclusions:**

While the acronym did not improve overall clinical performance, it was associated with a reduced intrinsic cognitive load in less experienced students. Introducing structured clinical reasoning tools earlier in the curriculum may help learners process clinical scenarios more effectively. Timing appears to be an important factor in the success of such interventions and should be considered in curriculum development.

**Supplementary Information:**

The online version contains supplementary material available at 10.1186/s12909-026-09639-0.

## Introduction

Clinical reasoning (CR) is defined as the complex process of collecting, integrating, and interpreting clinical knowledge to reach an appropriate diagnosis, initiating treatment, and providing effective patient management [1, 2]. It is a key competency for healthcare providers, particularly in high-stress fields such as anaesthesia and emergency medicine, where rapid decision-making can significantly affect patient outcomes [3]. Despite its importance, CR is often taught implicitly within medical curricula, with limited structured training opportunities [4].

Given its direct impact on patient safety, there is increasing consensus that CR should be taught explicitly using structured pedagogical approaches similar to those used for procedural skills training such as cardiopulmonary resuscitation [5–8].

Clinical reasoning involves an interaction between intuitive and analytical processes, as described by dual process theory. Intuitive (System 1) reasoning is fast and efficient but may be prone to cognitive biases, particularly in novice learners, whereas analytical (System 2) reasoning is slower, more deliberate, and potentially more accurate but difficult to sustain in time-pressured clinical environments [5, 9, 10]. Novices, including medical students and early trainees, are therefore particularly vulnerable to cognitive overload, which may contribute to delayed decision-making and diagnostic error [9, 10].

Cognitive load is defined by the American Psychological Association as the mental resources required to perform a task. In complex clinical situations, novices often rely on analytical processing, which can increase cognitive demand and reduce decision efficiency [11–13].

Non-technical skills (NTS) are integral to clinical performance and patient safety. These include cognitive, social, and personal resource management skills that complement technical abilities in high-risk, team-based environments such as emergency care [14, 15]. The Anaesthesia Non-Technical Skills (ANTS) framework is a validated tool used to assess decision-making, situational awareness, task management, and teamwork [16]. These domains are closely aligned with clinical reasoning processes in practice.

Cognitive load theory (CLT) proposes that learning is influenced by the limited capacity of working memory and the manner in which information is presented. Schemas facilitate learning by organising information into meaningful structures, thereby reducing cognitive burden and enhancing long-term retention [12, 17]. CLT distinguishes between intrinsic, extraneous, and germane cognitive load [18, 19]. Intrinsic load reflects the inherent complexity of the task and learner expertise; extraneous load arises from suboptimal instructional design and should be minimised; and germane load refers to cognitive resources dedicated to schema construction and learning. Effective instructional design aims to reduce extraneous load and optimise germane load to support learning.

Although CR is increasingly recognised as a skill that should be explicitly taught, there is ongoing debate regarding optimal teaching strategies and timing [20]. Structured cognitive aids may support cue recognition and decision-making under pressure, particularly for novice learners [5]. Heuristic approaches, including the use of acronyms, represent one such strategy to support clinical reasoning.

Acronyms have long been used as cognitive aids in medical education, enabling efficient recall of complex information, particularly in acute care settings [21, 22]. The ABCDE approach (Airway, Breathing, Circulation, Disability, Exposure), endorsed by the European Resuscitation Council, is a widely used example associated with improved patient outcomes in emergency care [23, 24]. Building on evidence supporting heuristic-based approaches to clinical reasoning [22], we developed the PAPER acronym through a modified Delphi process with clinical experts.

The acronym was subsequently piloted in simulation-based training with medical students in the 4th clinical semester and final year (Practical Year, PJ) at the University of Duisburg-Essen, Germany [25].

This study aimed to (a) develop a structured acronym-based tool to support clinical reasoning in emergency situations, (b) evaluate its impact on cognitive load in a simulation-based learning environment, and (c) explore the optimal timing of implementation within the medical curriculum. Specifically, we investigated the effects of the PAPER heuristic on clinical performance, non-technical skills, and cognitive load across two levels of medical training.

## Methods

### Study aims

The primary aim of this study was to develop and pilot an acronym-based heuristic to support clinical reasoning (CR) in emergency situations, with a particular focus on its impact on clinical performance. Secondary aims included evaluating its influence on non-technical skills in anaesthesia, assessing its effect on cognitive load (CL) during simulation-based training, and exploring the optimal timing for integrating a CR-focused intervention into the undergraduate medical curriculum.

### Developing the PAPER acronym

We used the Delphi method [26] as a multistage survey technique designed to achieve expert consensus on key elements of clinical reasoning (CR) in emergency contexts. This method enables participants to identify patterns, provide structured feedback, and converge toward agreement over successive rounds. Our Delphi process comprised three rounds:

Round 1: A working group composed of three members, an anaesthesiologist, a psychologist and nursing professional, and a medical student and paramedic, identified essential components of CR in emergency situations. These points were categorised on the basis of clinical relevance, and the PAPER acronym was established on the basis of the respective content and applicability in an emergency.

Round 2: A preliminary version of the PAPER schema was distributed via an electronic questionnaire to interdisciplinary acute care specialists in emergency medicine, anaesthesiology, and trauma surgery at University Hospital Essen, Germany. A total of *18* experts participated in the Delphi process until a consensus was reached. The participants anonymously rated each element of the acronym and provided qualitative feedback.

Round 3 involved analysing the aggregated ratings and comments. Therefore, the rankings for each acronym element were evaluated via a 10-point ordinal scale, with the results analysed via medians and interquartile ranges (IQRs). A final consensus version of the acronym was established on the basis of the results, and no further Delphi rounds were needed.

### Randomised single-blind pilot evaluation of the PAPER acronym

The PAPER acronym was evaluated for medical students participating in emergency simulation training. Two cohorts were included: students in the 4th clinical semester and those in the final year of medical training (11th semester; practical year, PJ). These groups were selected to assess clinical reasoning (CR) skills at different stages of undergraduate medical education.

**Fig. 1:**
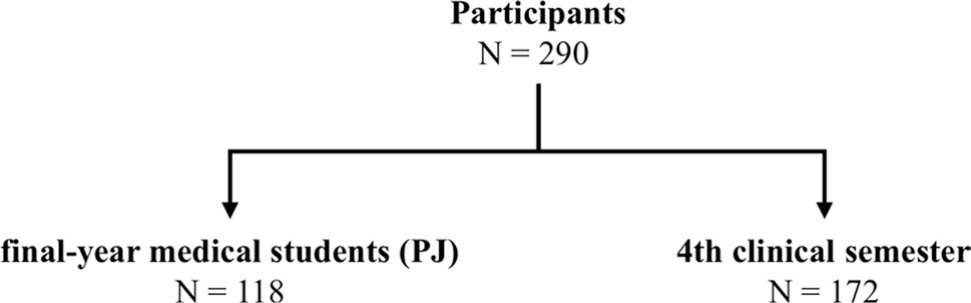
Distribution of participants across semesters

A total of *N* = 290 medical students from the 4th clinical semester and the Practical Year (PJ; 11th semester) were included in the study. Participants were enrolled in courses conducted during the summer term of 2021 and the winter term of 2021–2022 and were randomly allocated into control and intervention groups (Fig 1).

The control groups received standard simulation-based training. The intervention groups received an additional 20-minute lecture introducing the concept of clinical reasoning (CR), its relevance for patient safety, and its potential impact on clinical outcomes. The PAPER acronym was presented during the lecture and explained as a structured tool to support CR in emergency situations.

For 4th-semester students, the lecture was delivered at the beginning of a two-week practicum in the Department of Anaesthesiology and Intensive Care Medicine one day prior to the simulation sessions. These students then participated in two consecutive mornings of simulation training, covering nine emergency scenarios over the course of one week. The PJ semester students received the lecture immediately before starting the simulation training, followed by nine two-hour simulation sessions over nine weeks. This slight variation in the PAPER introduction was unavoidable because of the schedule of training seminars. In both groups, the simulations covered various emergency scenarios, including resuscitation, haemorrhagic shock, and acute abdomen (Table 1).

**Table 1:** List of cases used in simulation training

Field	Description of the scenario
Anaesthesiology and Emergency Medicine	Cardiac Arrest
Vascular Surgery	Haemorrhagic shock of a postoperative patient
Urology	Urological Emergency
Visceral Surgery	Acute abdomen
Otorhinolaryngology	Epistaxis
Paediatrics	Acute foreign body aspiration
Acute General Medicine	unclear loss of consciousness after a fall
Trauma Surgery	Neck of Femur Fracture
Acute General Medicine	Acute Dyspnoea

Each simulation was evaluated using the Questionnaire of Clinical Decision Making, a 10-item checklist, specifically developed to assess observed clinical performance in simulation-based scenarios. The items were designed to reflect core components of clinical decision-making, with reference to structured acronym-based approaches. Although the questionnaire was specifically developed for this study, a formal validation process has been conducted and submitted for publication. Preliminary analyses indicate acceptable internal consistency and provide initial evidence supporting construct content, and face validity. The instrument was therefore used as an exploratory measure of observed clinical performance in simulation-based scenarios. English-language version of the questionnaire is available as a supplementary file (see Supplementary Material 1). These parameters serve as indicators of clinical reasoning (CR) performance within the broader context of clinical management and are therefore used to operationalise CR in the simulation setting [27, 28]. Nontechnical skills were assessed via the Anaesthetists’ Nontechnical Skills (ANTS) system, which covers the four behavioural categories mentioned above [29]. Evaluations were conducted by senior physicians who were blinded to group allocation but aware of the participants’ semester level. After each simulation, participants completed a standardised and validated 20-item cognitive load questionnaire, which assessed three dimensions of cognitive load: intrinsic, extraneous, and germane load [19]. All the students received video-assisted feedback on their performance.

Incomplete checklists, ANTS ratings, or cognitive load questionnaires were excluded from the final analysis.

The simulations were conducted in groups of two to three students. Clinical performance and nontechnical skills (ANTS) were assessed at the group level by experienced clinical raters. In contrast, cognitive load was assessed individually by each student, resulting in a greater number of responses for the cognitive load data than for the ANTS and clinical performance ratings.

### Data analysis

Statistical analyses were conducted via IBM SPSS Statistics (version 28.0; IBM Corp., Armonk, NY, USA). The effects of PAPER acronym use on outcomes were analysed via a two-way factorial ANOVA (2 × 2 design) with the between-group factors PAPER intervention (PAPER versus control) and semester affiliation (4th clinical semester vs. final-year [PJ] students). The design allowed us to test the effects of the PAPER intervention across semester groups (i.e., overall differences between the PAPER intervention and control groups), the effects of the semester (i.e., differences between lower- and higher-semester students), and the interaction effect of the PAPER intervention (i.e., whether the impact of the intervention varied by educational stage). When significant ANOVA effects were found, post hoc pairwise comparisons were performed using Bonferroni-corrected independent-samples t-tests. Statistical significance was set at *p* < 0.05.

The study involved the evaluation of educational outcomes across two academic terms. Ethical approval for the study was obtained from the Ethics Committee of the University of Duisburg-Essen. All the participating medical students provided written informed consent prior to participation. Participation was voluntary, and all the data were collected anonymously. Students were randomly assigned to either the clinical reasoning (CR) intervention or a standard teaching control group.

## Results

### Final version of the PAPER Acronym

**Fig. 2:**
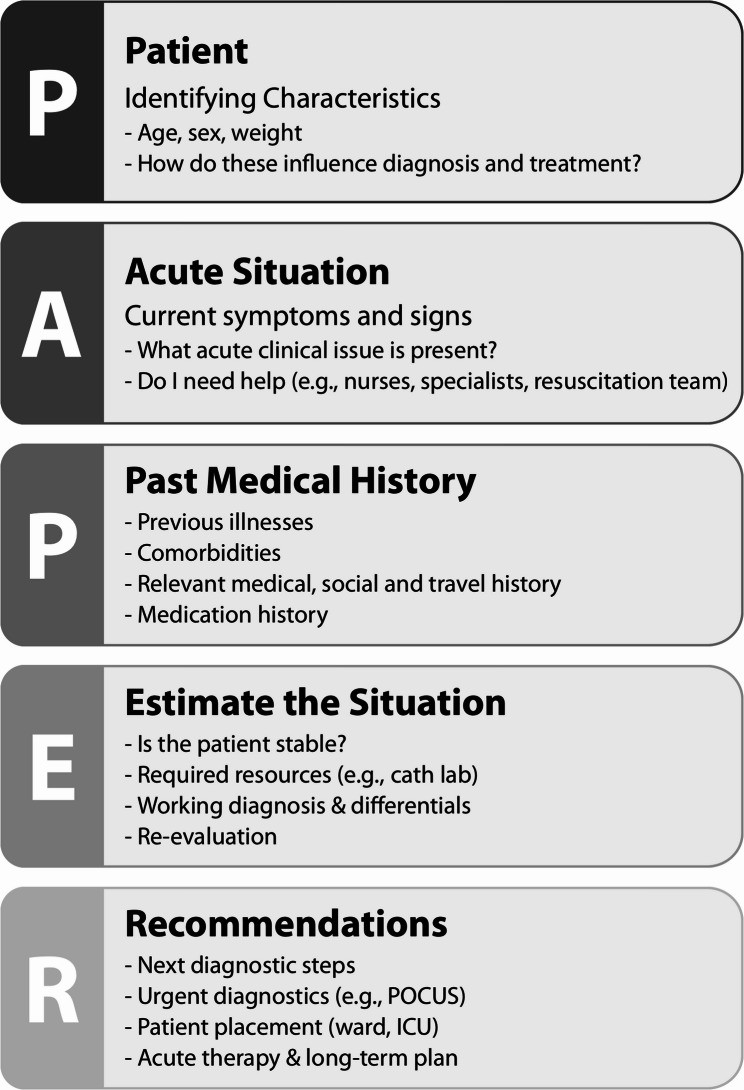
Final version of the PAPER acronym

The final round of the Delphi process resulted in the development of the PAPER acronym, which stands for patient, details of the acute situation, relevant past medical history, estimation of the situation, and recommendations (Fig. 2).

### Demographics

The study sample was predominantly female (61.8%), with a mean age of 24.8 years across all groups. The cohorts were relatively homogeneous both within and across semester levels. The final number of analysed questionnaires is presented in Table 2.

**Table 2:** Distribution of the total evaluated cognitive load (CL) questionnaires, assessment questionnaires for observed clinical performance (CP), and ANTS questionnaires regarding semester affiliation, as well as the control and intervention groups

	Control groups			Intervention groups (PAPER)			∑
	4th clin. Sem.	PJ	Total	4th clin. Sem.	PJ	Total	
**CL**	199	45	244	213	56	269	513
**CP**	72	18	90	86	22	108	198
**ANTS**	70	17	87	83	21	104	191

Assessments of observed clinical performance (CP) and nontechnical skills (ANTS) were conducted at the group level by trained raters, with each simulation group consisting of approximately three students. In contrast, cognitive load (CL) was assessed individually by each student participant via a validated self-report questionnaire. This explains the greater number of CL questionnaires compared with the CR and ANTS assessments.

### Effects of PAPER and semester affiliation on the evaluation of Clinical Performance (CP)

**Fig. 3:**
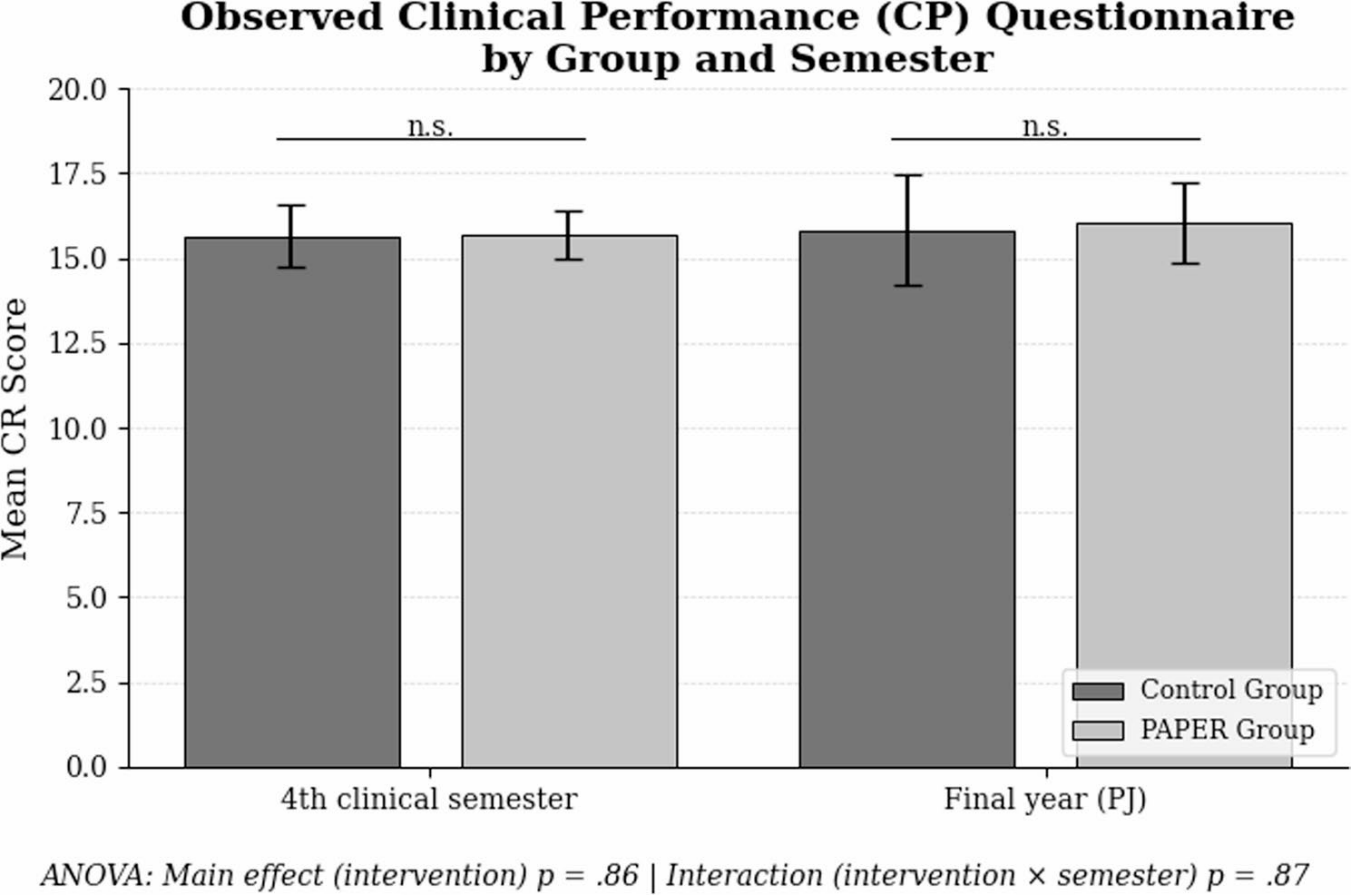
Mean values for the observed clinical performance of the 4th clinical semester students and the PJ students in the PAPER intervention and control groups

The use of the PAPER acronym did not result in a significant difference in clinical performance (i.e., CRP scores during simulations) between the control and intervention groups (F_(1, 194)_ = 0.03, *p* = 0.86, n_p_^2^ = 0.00, ANOVA main effect of intervention), with no differential effects within the two semester groups (F_(1, 194)_ = 0.03, *p* = 0.87, n_p_^2^ = 0.00; ANOVA intervention × semester interaction). In addition, the CP scores of students in the 4th clinical semester and PJ students did not differ (F_(1, 194)_ = 0.21, *p* = 0.65, n_p_^2^ = 0.01, ANOVA main effect of semester affiliation) (Fig. 3).

### Effects of PAPER and semester affiliation on the assessment of ANTS

The assessment of ANTS, including situational awareness, decision-making, task management, and teamwork, was analysed to determine whether there were any differences between the PAPER interventions across and within semesters (Table 3).

**Table 3:** ANTS scores for students in the 4th clinical semester and PJ students in the control and PAPER intervention groups. The data are shown as the means ± standard deviations

	Control groups		Intervention groups (PAPER)	
	4th semester	PJ	4th semester	PJ
Situational Awareness	9.06 (± 2.61)	10.06 (± 2.73)	9.08 (± 2.36)	9.71 (± 2.51)
Decision-Making	5.40 (± 1.84)	6.71 (± 1.61)	5.61 (± 1.90)	5.86 (± 1.96)
Task Management	13.47 (± 5.48)	16.65 (± 4,00)	14.70 (± 4.43)	14.95 (± 5.05)
Teamwork	5.93 (± 2,12)	7.06 (± 1,68)	6.25 (± 1,75)	6.62 (± 1.77)

Teamwork: There was no significant difference in teamwork scores between the intervention and control groups (F_(1, 187)_ = 0.028, *p* = 0.867, n_p_^2^ = 0.00, ANOVA main effect of intervention), nor was there an interaction effect between intervention and semester affiliation (F_(1, 187)_ = 1.24, *p* = 0.27, n_p_^2^ = 0.01, ANOVA intervention × semester interaction effect). However, independent of the PAPER intervention, decision-making differed significantly between students in the 4th clinical semester and PJ students (F_(1, 187)_ = 4.73, *p* = 0.03, n_p_^2^ = 0.03, ANOVA main effect of semester affiliation), with the older students showing improved scores.

Situational Awareness: The PAPER intervention led to no significant difference between the control and intervention groups (F_(1, 187)_ = 0.12, *p* = 0.73, n_p_^2^ = 0.01, ANOVA main effect of intervention). No significant interaction effect between intervention and semester affiliation (F_(1, 187)_ = 0.02, *p* = 0.68, n_p_^2^ = 0.01) or difference between semester groups was observed (F_(1, 187)_ = 3.20, *p* = 0.08, n_p_^2^ = 0.02, ANOVA main effect of semester affiliation).

Decision-Making: The PAPER intervention did not induce a difference in decision-making between the control and intervention groups (F_(1, 187)_ = 0.88, *p* = 0.35, n_p_^2^ = 0.01, ANOVA main effect of intervention; F_(1, 187)_ = 2.42, *p* = 0.12, n_p_^2^ = 0.01; ANOVA interaction effect). However, independent of the PAPER intervention, decision-making differed significantly between students in the fourth clinical semester and PJ students (F_(1, 187)_ = 5.22, *p* = 0.02, n_p_^2^ = 0.03, ANOVA main effect of semester affiliation), with the older students showing improved scores.

Task Management: The PAPER intervention did not lead to differences in decision-making between the control and intervention groups (F_(1, 187)_ = 0.07, *p* = 0.79, n_p_^2^ = 0.00, main effect of intervention). No evidence for an interaction of intervention and semester affiliation (F_(1, 187)_ = 2.7, *p* = 0.10, n_p_^2^ = 0.01, ANOVA interaction effect) or a difference between semester groups (F_(1, 187)_ = 3.72, *p* = 0.06, n_p_^2^ = 0.02, ANOVA main effect of semester affiliation) was observed.

### Effects of PAPER and semester affiliation on cognitive load

**Fig. 4:**
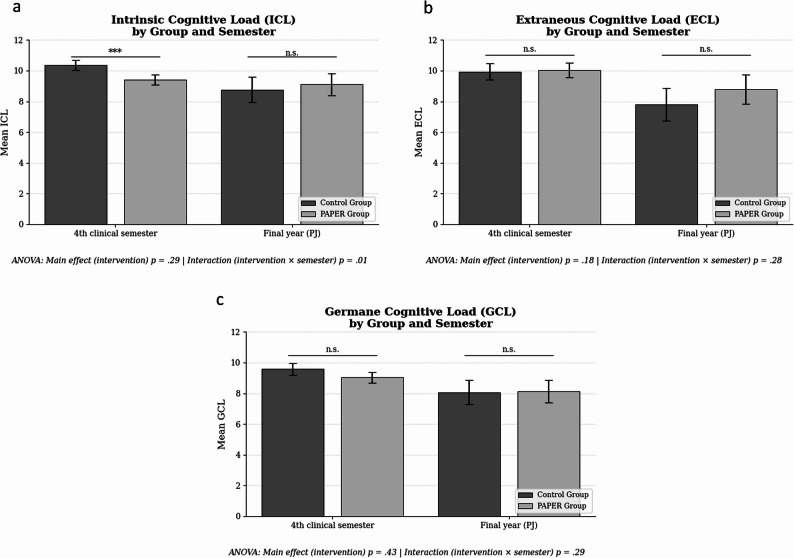
Figures 4**a**, 4**b**, 4**c**: Mean values for the cognitive load of students in the 4th clinical semester and PJ students in the PAPER intervention and control groups, shown for the subscales intrinsic cognitive load (ICL, 4**a**), extraneous cognitive load (ECL, 4**b**), and germane cognitive load (GCL, 4**c**)

For intrinsic cognitive load (ICL) scores, no significant difference between the control and intervention groups was observed (F_(1, 509)_ = 1.10, *p* = 0.29, n_p_^2^ = 0.00, ANOVA main effect of intervention). Interestingly, the analysis revealed a significant interaction effect between the intervention and semester affiliation (F _(1, 509)_ = 5.24, *p* = 0.02, n_p_^2^ = 0.01, ANOVA interaction effect), suggesting that the intervention had a differential effect on the two semester groups. Indeed, post hoc testing indicated a lower ICL score in the intervention group than in the control group within the 4th clinical semester (t _(410)_ = 3.85, *p* < 0.001). In contrast, no intervention effect was found for PJ students (t _(99)_ = -0.65, *p* = 0.52) (Fig. 4a). Independent of the intervention, PJ students had lower overall ICL scores than did students in the 4th clinical semester did (F_(1, 509)_ = 11.22, *p* < 0.001, n_p_^2^ = 0.022, main effect of semester affiliation).

PAPER intervention did not affect extraneous cognitive load (ECL). Analysis revealed no significant difference between the control and intervention groups (F _(1, 509)_ = 1.83, *p* = 0.18, n_p_^2^ = 0.04, ANOVA main effect of intervention) or an interaction of intervention and semester affiliation (F _(1, 509)_ = 1.16, *p* = 0.28, n_p_^2^ = 0.002). Independent of the intervention, PJ students reported significantly lower ECL scores than did students in the 4th clinical semester did (F_(1, 509)_ = 17.47, *p* < 0.01, n_p_^2^ = 0.033, main effect of semester affiliation) (Fig. 4b).

Finally, the PAPER intervention did not change the GCL scores. The data revealed no significant difference between the control and PAPER intervention groups (F _(1, 509)_ = 0.635, *p* = 0.43, n_p_^2^ = 0.001, main effect intervention) or between the intervention and semester affiliation groups (F_(1, 509)_ = 1.10, *p* = 0.29, n_p_^2^ = 0.002). Independent of the PAPER intervention, significantly lower GCL scores were observed in PJ students than in students in the 4th clinical semester (F_(1, 509_ = 16.20, *p* < 0.001, n_p_^2^ = 0.031) (Fig. 4c).

## Discussion

### Non-technical skills, CR, and the PAPER acronym

This study examined the impact of a newly developed PAPER acronym on clinical performance during simulated emergency medical scenarios. The acronym was created through a Delphi procedure involving medical experts with broad experience in acute care specialties such as anaesthesiology and emergency medicine. Despite this structured development, the intervention did not result in a statistically significant improvement in clinical performance compared with that of the control group. No significant differences were found either between the intervention and control groups or between the two student cohorts (4th clinical semester vs. PJ students). These findings suggest that the introduction of the PAPER acronym did not substantially alter the students’ clinical reasoning or clinical decision-making performance within the simulation-based learning context. Several factors may account for this lack of measurable effects.

Boshuizen & Schmidt [30] suggested that novice medical students typically possess limited clinical decision-making skills, implying that clinical reasoning (CR) is a competence acquired progressively through experience [10]. Accordingly, the learning objectives of the simulation were adapted to match the expected performance levels of the respective student cohorts. Although the raters were blinded to group allocation (intervention vs. control), they were aware of the students’ semester affiliation. This awareness may have introduced a leniency bias, with raters potentially being more forgiving of errors made by less experienced students, thereby slightly inflating performance scores in that group. Alternatively, the similar clinical performance scores observed across cohorts might indicate that the learning objectives were appropriate for each level of training and were successfully achieved.

It remains unclear whether an alternative timing of PAPER intervention might have enhanced its effectiveness. In the present study, the PJ intervention group received a 20-minute seminar introducing the acronym and its intended use immediately before the simulation. This brief interval may not have allowed sufficient time for the students to internalise and apply the concept effectively [30]. Clinical reasoning is a dynamic, experience-dependent skill shaped not only by clinical exposure but also by individual interpretation of clinical experiences [4]. It is conceivable that more advanced students had already developed entrenched learning strategies, making them less receptive to newly introduced cognitive tools [31].

Introducing a CR seminar earlier in the curriculum, before the fourth clinical semester, may be a more effective approach. Earlier exposure would provide students with more time to integrate CR strategies into their learning routines, facilitating deeper cognitive embedding. Clinical performance is a complex, multifaceted skill involving clinical knowledge as well as factors such as group dynamics and individual competence. These variables are inherently difficult to isolate and quantify in simulation-based educational research, which may contribute to the absence of significant group differences observed in this study. A further methodological consideration relates to the assessment of clinical reasoning using a de novo questionnaire developed for this study. The instrument was designed to capture key observable components of clinical decision-making in a simulation context and was explicitly constructed to reflect core elements of the construct, thereby supporting its face validity and theoretical alignment with established models of clinical reasoning. Subsequent psychometric evaluation has been conducted, with results supporting acceptable measurement properties and providing evidence for construct validity; these findings have been submitted for publication. Accordingly, while formal validation was not completed prior to study implementation, the instrument was not intended to capture the full cognitive reasoning process, but rather the externalised, observable manifestations of clinical decision-making. This distinction is particularly relevant, as clinical reasoning is a multidimensional construct that includes data gathering, hypothesis generation, synthesis, and decision-making processes that may not be fully captured through checklist-based assessment tools. Therefore, the present findings should be interpreted as reflecting differences in observed clinical performance within a simulated environment rather than definitive changes in internal clinical reasoning competence. Future studies should incorporate validated instruments or multi-method approaches to more comprehensively assess clinical reasoning processes.

Although the PAPER acronym shares structural similarities with established communication frameworks such as SBAR, its intended purpose differs fundamentally. SBAR is primarily designed as a structured communication tool to standardise information transfer between healthcare professionals, particularly in handover situations. In contrast, PAPER was developed as a cognitive heuristic to support individual clinical reasoning during acute patient assessment and decision-making. Rather than standardising communication output, PAPER aims to structure the internal cognitive processes involved in early problem representation, information prioritisation, and situational interpretation. Therefore, the contribution of PAPER lies not in improving communication per se, but in providing a scaffold for novice learners to organise clinical information during the reasoning process itself. This distinction is particularly relevant when considering its application in undergraduate medical education, where learners are still developing internal cognitive schemas for clinical decision-making.

### Impact of ANTS and PAPER

The effect of the PAPER acronym on anaesthesia-related nontechnical skills (ANTS) during simulated emergency scenarios was investigated in this study. No significant differences were observed between the intervention and control groups within each semester cohort, particularly in the situational awareness and task management categories. However, PJ students demonstrated significantly higher clinical-reasoning and teamwork scores than their junior counterparts did, irrespective of the intervention. This finding aligns with expectations, as more advanced students are likely to benefit from greater clinical exposure and a more developed working memory capacity, which supports complex cognitive tasks such as clinical reasoning.

While these improvements in nontechnical skills did not translate into significantly better clinical performance scores, they suggest that implicit learning processes may occur between the fourth clinical semester and the final year of medical training. The observed gains are likely attributable to accumulated clinical experience, enabling students to observe, reflect on, and internalise effective decision-making behaviours and teamwork strategies.

These results underscore the importance of appropriately timing the introduction of explicit clinical reasoning (CR) instruction in the medical curriculum. CR teaching should ideally begin before the fourth clinical semester, when students are still shaping their cognitive frameworks, but after they have gained sufficient clinical exposure to contextualise and apply these concepts meaningfully. Embedding CR as a longitudinal, explicitly taught competency may enhance its integration into future clinical performance.

### Cognitive load theory and PAPER acronym intervention: role of the PAPER acronym in reducing cognitive load

The application of structured frameworks, such as the PAPER acronym, has the potential to reduce the cognitive load by streamlining information processing and retrieval, thereby supporting clinical reasoning (CR). In clinical settings, cognitive load is a critical factor influencing the quality of decision-making, a central element of clinical reasoning [32]. A tool such as the PAPER scheme could reduce intrinsic cognitive load (ICL), foster learning and retention, and result in more effective outcome reasoning. To evaluate this potential, cognitive load was assessed as a proxy indicator for CR effectiveness. Notably, the ICL scores were significantly lower among the 4th clinical semester students in the PAPER intervention group than in those in the control group. This finding suggests that the PAPER intervention had a beneficial effect on cognitive processing in less experienced learners. Since the ICL reflects the inherent complexity of a task independent of the instructional format, a reduction in the ICL implies that the acronym may have enabled students to mentally organise relevant clinical information into more manageable, coherent units, facilitating learning while decreasing the effort required for information processing [31, 33]. These findings are consistent with cognitive load theory, which posits that reducing unnecessary cognitive burden can enhance learning and problem-solving performance. The results therefore indicate that structured support tools such as PAPER may be particularly valuable in the early stages of clinical education, where cognitive resources are limited and still under development.

This effect was not observed in PJ students, likely because of their more advanced clinical knowledge and experience, which may have reduced the relative benefit of the intervention. Additionally, the timing and structure of the PJ curriculum, specifically, the delivery of the PAPER intervention immediately prior to simulation training, may have limited the opportunity to integrate the acronym into working memory and apply it meaningfully during the scenarios. As hypothesised, the PAPER intervention did not significantly affect extraneous cognitive load (ECL), suggesting that the instructional format did not introduce unnecessary complexity. Similarly, no significant differences were observed for germane cognitive load (GCL) between the intervention and control groups, indicating that the acronym did not alter the extent to which participants engaged in meaningful learning processes across cohorts. Germane cognitive load is considered a beneficial component of cognitive load theory, as it reflects mental effort directed toward schema construction, knowledge organisation, and metacognitive processing that facilitates long-term learning.

We hypothesised that the PAPER intervention would have a greater impact on students who depend more significantly on structured frameworks to process information. Consistent with this assumption, students in the earlier stages of medical education, specifically, those in the 4th clinical semester, exhibited a generally greater cognitive load across all three dimensions (intrinsic, extraneous, and germane cognitive load) than PJ students did. Interestingly, the germane cognitive load (GCL) was significantly greater among fourth-semester students, suggesting a greater level of cognitive engagement in processing and integrating new information. Novice learners typically approach clinical problems through hypothetical-deductive reasoning strategies, which, owing to limited prior knowledge, may increase susceptibility to cognitive overload [10]. Their reduced situational awareness and less developed mental schemas further contribute to higher cognitive demands. Notably, for many 4th-semester participants, this simulation represented their first formal exposure to both high-fidelity simulation and structured clinical reasoning tools such as the PAPER acronym. This confluence of unfamiliar educational formats and content complexity may have overwhelmed both the intervention and control groups. It is therefore possible that the intervention’s effects were diluted by this cognitive burden and that detecting significant group differences would require a substantially larger sample size.

The finding that more advanced students generally exhibited a lower cognitive load, regardless of the use of structured tools such as the PAPER acronym, aligns with the notion that increased clinical experience and more developed working memory enable them to manage complex cognitive demands more efficiently. The overall reduction in cognitive load observed in more advanced students does not imply unused or “freed” cognitive capacity but rather reflects the development of automated schemas and more efficient cognitive processing strategies, which reduce working memory demands during clinical reasoning tasks. In contrast, the significantly greater germane cognitive load (GCL) observed in 4th-year clinical-semester students may reflect the increased mental effort required to construct and consolidate new cognitive schemas. This is particularly noteworthy given the concurrently lower intrinsic cognitive load (ICL) in the intervention group, which suggests that the acronym may have facilitated more efficient processing of the clinical scenario. The increase in GCL, despite the reduction in ICL, may indicate active engagement with the tool as a learning strategy, supporting the hypothesis that structured cognitive aids can promote deeper processing in novice learners. However, the effectiveness of such interventions is likely contingent upon appropriate timing within the curriculum. Introducing the acronym before students have developed sufficient foundational knowledge and cognitive structures may limit its utility. Therefore, structured tools such as the PAPER acronym should ideally be implemented at a point when learners are cognitively prepared to integrate and apply them within the dynamic context of clinical decision-making.

Students, although it did not yield a statistically significant effect on germane cognitive load (GCL). Nevertheless, the observed trends suggest increased engagement with acronyms among less experienced students. Furthermore, semester affiliation plays a critical role in shaping students’ cognitive load profiles, highlighting the relevance of learner experience in moderating the impact of instructional interventions. These findings underscore the importance of differentiating between distinct types of cognitive load when evaluating the efficacy of clinical reasoning (CR) training tools. Importantly, the results suggest that the optimal window for implementing explicit CR instruction is prior to students’ entry into clinical care environments [28]. Early integration of structured decision-making strategies would enable learners to apply these principles during both simulated and real patient encounters. By the practical year (PJ), students may have already developed individualised approaches to clinical reasoning, potentially limiting the effectiveness of newly introduced frameworks such as the PAPER acronym.

### Limitations of the study

One limitation of this study concerns the simplified nature of the simulation scenarios, which may not adequately reflect the complexity of real-world clinical reasoning (CR). The simulations were intentionally mono-factorial, centered around a single diagnosis and structured in a linear sequence, history-taking, examination, diagnosis, and treatment. This design aimed to reduce cognitive overload and facilitate learning by encouraging a hypothetical-analytical approach. While pedagogically justified, such simplification likely contributed to the overall high clinical performance scores and the absence of significant differences between the intervention and control groups [34]. In contrast, real-life clinical encounters are often multifactorial, ambiguous, and dynamic, making extraneous cognitive load (ECL) a more salient factor. In such settings, the use of structured decision aids such as the PAPER acronym might prove more beneficial. Future research employing more complex, realistic simulations could yield different insights, particularly for more experienced learners. Importantly, the effectiveness of the PAPER scheme was assessed in a controlled simulation environment; its utility in real-world acute care settings, where decision-making is influenced by numerous unpredictable variables, remains to be investigated.

A further limitation lies in the timing of the PAPER acronym intervention. While younger students appeared more responsive to the acronym, older students, likely having already developed personal CR strategies, were less affected. The PJ students received a 20-minute introduction to the acronym immediately prior to the simulation, leaving minimal opportunity for integration into their cognitive framework. In contrast, 4th-semester students received the same instructional session at the beginning of a two-week practicum, allowing up to two days for reflection and retention. This difference in instructional timing, dictated by course logistics, may have contributed to the divergent outcomes and highlighted crucial considerations for curriculum design: structured CR training should ideally be introduced before students internalise their own reasoning patterns. Earlier implementation could improve the uptake and effectiveness of tools such as the PAPER acronym.

## Conclusions

Structured decision aids such as the PAPER acronym appear particularly beneficial when introduced before students are exposed to clinical environments. The findings of this study suggest that explicit instruction in clinical reasoning (CR) as a core medical competency, along with the use of structured learning tools, should be implemented early in the curriculum, preferably prior to first clinical placements. Early exposure may provide students with a conceptual framework that facilitates the application of CR skills in increasingly complex clinical contexts as their training progresses. While the intervention produced mixed results, this outcome likely reflects the inherent complexity of clinical reasoning itself and underscores the importance of timing in the design of CR training. Further research is warranted to determine the most effective timing, content, and instructional formats for integrating CR education into undergraduate medical training.

## Supplementary information

Supplementary Material 1.

## Data Availability

The datasets used and/or analysed during the current study are available from the corresponding author upon reasonable request.

## Glossary

CR: Clinical reasoning
ECL: Extraneous cognitive load
GCL: Germane cognitive load
ICL: Intrinsic cognitive load
PAPER: Patient, details of acute situation, relevant medical history, estimate the situation, recommendations
