# Supplementary material for: The PAPER heuristic: a structured cognitive aid to reduce intrinsic cognitive load in early clinical reasoning training
Source: BMC Med Educ. 2026 Jun 16;26:1007. doi: 10.1186/s12909-026-09639-0 (PMC13282858; doi:10.1186/s12909-026-09639-0)
Supplement: Supplementary file 1 — Supplementary Material 1. [file 12909_2026_9639_MOESM1_ESM.pdf]

Questionnaire of Clinical Decision Making

(*Supplementary Material 1 – English Version*)

Checklist: **Evaluation of students in the application of a structured acronym to support clinical decision-making**

Case:
Tutor:
Date:

During the simulation, participants are observed with regard to the categories listed below. Please tick one box for each category.

No.	Item	Correctly performed	Minor errors	Incorrectly performed
		**2 Pts**	**1 Pts**	**0 Pts**
1.	Student introduces themselves and identifies the patient.	☐	☐	☐
2.	Student correctly assesses the acute situation.	☐	☐	☐
3.	Student obtains a relevant medical history.	☐	☐	☐
4.	Student evaluates the patient's status.	☐	☐	☐
5.	Student performs an appropriate physical examination.	☐	☐	☐
6.	Student presents an appropriate primary diagnosis.	☐	☐	☐
7.	Student orders appropriate diagnostic procedures.	☐	☐	☐
8.	Student seeks appropriate help or support.	☐	☐	☐
9.	Student initiates basic therapeutic interventions.	☐	☐	☐
10.	Student outlines a plan for further management.	☐	☐	☐
	**Total score:**	______ / 20 pts		

Thank you for your participation!
